# Protective Capacity of Statins during Pneumonia Is Dependent on Etiological Agent and Obesity

**DOI:** 10.3389/fcimb.2018.00041

**Published:** 2018-02-15

**Authors:** Erik A. Karlsson, Stacey Schultz-Cherry, Jason W. Rosch

**Affiliations:** Department of Infectious Diseases, St. Jude Children's Research Hospital, Memphis, TN, United States

**Keywords:** *Streptococcus pneumoniae*, influenza, statin, obesity, pneumonia

## Abstract

Acute respiratory infections are a leading cause of death worldwide. Clinical data is conflicted regarding whether statins improve outcomes for pneumonia. Potential confounding factors including specific etiology of pneumonia as well as obesity could potentially mask protective benefit. Obesity is a risk factor for high cholesterol, the main target for statin therapy. We demonstrate that statin intervention conferred no protective benefit in the context of wild-type mice regardless of infectious agent. Statin intervention conferred either a protective benefit, during influenza infection, or detrimental effect, in the case of pneumococcal infection, in obese animals. These data suggest etiology of pneumonia in the context of obesity could be dramatically altered by the protective effects of statin therapy during bacterial and viral pneumonia.

## Introduction

Statins (3-hydroxy-3-methylglutaryl coenzyme A reductase inhibitors) lower circulating lipid levels and decrease risk of cardiovascular disease. Statins also have a number of pleiotropic effects including anti-inflammatory and immunomodulatory properties that may confer benefit during infection (Jain and Ridker, 2005; Fedson, 2006). Several retrospective clinical studies have attempted to observe the beneficial effects of statins in regards to infection with conflicting conclusions (Mortensen et al., 2005, 2008, 2012; van de Garde et al., 2006; Frost et al., 2007; Schlienger et al., 2007; Chalmers et al., 2008; Thomsen et al., 2008; Dublin et al., 2009; Myles et al., 2009; Douglas et al., 2011; Vinogradova et al., 2011; Yende et al., 2011; Nielsen et al., 2012; Novack et al., 2012; Rothberg et al., 2012; Doshi et al., 2013). The majority of these studies suggest a beneficial effect (Mortensen et al., 2005, 2008, 2012; van de Garde et al., 2006; Frost et al., 2007; Schlienger et al., 2007; Chalmers et al., 2008; Thomsen et al., 2008; Myles et al., 2009; Douglas et al., 2011; Vinogradova et al., 2011; Nielsen et al., 2012; Novack et al., 2012; Rothberg et al., 2012; Doshi et al., 2013); however, several have found no difference or even a harmful effect of statins on pneumonia-related morbidity and mortality (Dublin et al., 2009; Yende et al., 2011). A majority of the studies do not control for pneumonia etiology based on laboratory-confirmed diagnosis (Mortensen et al., 2005, 2008, 2012; van de Garde et al., 2006; Schlienger et al., 2007; Chalmers et al., 2008; Thomsen et al., 2008; Dublin et al., 2009; Myles et al., 2009; Douglas et al., 2011; Vinogradova et al., 2011; Yende et al., 2011; Nielsen et al., 2012; Novack et al., 2012), potentially introducing variable effects of statins due to distinct pathogenic mechanisms of viral and bacterial agents. In addition, the majority of the studies examining the pleiotropic effects of statins usually control for risk factors, such as diabetes and obesity, without factoring them as a separate group that may have altered pathogenesis and course of acute respiratory infection.

It is becoming increasingly apparent that the metabolic status of the host can profoundly impact infection susceptibility as well as to decrease responses to vaccines. Given that in the United States alone, 68.0% of the population has a Body Mass Index (BMI) ≥ 25 kg/m^2^ indicating the majority of the population is overweight or obese, the role of obesity is a critical consideration (Flegal et al., 2010). Obesity has been found to be an independent risk factor for influenza severity and mortality, (Karlsson and Beck, 2010) findings effectively modeled in several mouse studies (Smith et al., 2007; Easterbrook et al., 2011; O'Brien et al., 2012). Obesity is also strongly associated with cardiovascular disease risk. Indeed, 80% of obese individuals exhibit classical metabolic lipid changes and statins are the drug of choice for lowering lipid levels (Tonstad and Despres, 2011). We hypothesized that metabolic status would profoundly impact the protective capacity of statins in the context of respiratory infection. Our data indicates that obesity as well as whether the pneumonia is bacterial or viral in origin as critical determinants for the protective effects of statins in the context respiratory disease.

## Methods

### Ethics statement

All experiments involving animals were performed with prior approval of and in accordance with guidelines of the St. Jude Institutional Animal Care and Use Committee. The St Jude laboratory animal facilities have been fully accredited by the American Association for Accreditation of Laboratory Animal Care. Laboratory animals are maintained in accordance with the applicable portions of the Animal Welfare Act and the guidelines prescribed in the DHHS publication, Guide for the Care and Use of Laboratory Animals.

### Bacterial and viral strains and growth conditions

The *Streptococcus pneumoniae* D39x (serotype 2) (Francis et al., 2001) pneumococcal strain was grown overnight at 37°C in a 5% CO_2_ humidified incubator after being inoculated onto tryptic soy agar (TSA) plates supplemented with 3% sheep blood. Strains were then inoculated directly into semisynthetic liquid culture (CY broth) and grown to log phase before being administered to mice. The influenza A virus A/California/04/2009(A/H1N1pdm) generated by reverse genetics (McAuley et al., 2007), was grown in Madin-Darby canine kidney (MDCK) cells.

### Mice utilized in cholesterol and obesity studies

Wild-type, female C57/Bl6 mice and genetically obese, B6.Cg-*Lep*°^*b*^/J (*ob/ob*) mice were obtained from The Jackson Laboratory, Bar Harbor, ME, USA. *ob/ob* mice lack the anorexigenic adipokine Leptin which makes them hyperphagic leading to profound obesity. These animals have been used extensively to study both diabetes and obesity phenotypes (Lutz and Woods, 2012).

### Statin treatment

Mice were maintained on a diet containing 120 ppm simvastatin (Diet #5053, Purina TestDiet) beginning at age 4 weeks and continuing for 4 weeks. Mock treated animals received a matched diet lacking simvastatin. Following treatment, serum was collected and high-density lipoprotein (HDL), low-density lipoprotein (LDL), triacylglycerol (TAG), and total cholesterol levels were measured to confirm pharmacological benefit. Mice were fed *ad libitum* and were maintained on the diet throughout the course of the infectious challenges.

### Cholesterol diet

Mice were placed for 4 weeks on either a high-cholesterol diet (HCD) (15.8% fat, 1.25% cholesterol, and 0.5% cholate, test diet #90221; Harlan Laboratories, Indianapolis, IN) or a normal diet (ND), which was identical, except for omission of cocoa butter, cholesterol, and cholate (test diet #95138). Cholesterol levels were measured in serum using the ABX Pentra Cholesterol CP kit (Horiba ABX, Montpellier, France) according to manufacturer guidelines.

### Mouse challenges

All mice were maintained in BSL2-level, specific pathogen free facilities. All experimental inoculation procedures were conducted under general anesthesia with inhaled isofluorane at 2.5%. Mice were monitored daily for signs of infection.

For bacterial burden and survival studies, bacterial strains were grown in C+Y media to an OD_620_ of 0.4 and diluted according to a previously determined standard curve. Bacteria were introduced into mice (Jackson Laboratory) via intranasal (IN) administration of 10^7^ CFU of bacteria in PBS (30 μL). Mice were monitored for disease progression and euthanized via CO_2_ asphyxiation. Blood for titer determination was collected via tail snip at 24 and 48 h post-infection and subsequent serial dilution and plating. For the viral challenge, mice were lightly anesthetized with isofluorane and intranasally inoculated with 10^2^ TCID_50_ units of influenza A/H1N1pdm in 25 μl PBS. Mice were monitored daily for clinical signs of infection (Morton, 2000) and weighed every 24 h post-inoculation (pi). Co-infection challenges were performed as previously described (Karlsson et al., 2017). Survival data were analyzed using the Mantel-Cox log rank test in Prism 6. Bacterial and viral titers were compared using non-parametric Mann-Whitney *t*-test in Prism 6.

### Viral titer determination

Viral titers from lung homogenates, BALF and nasal washes were determined by 50% tissue culture infectious dose (TCID_50_) on Manin Darby Canine Kidney (MDCK) cells (Cline et al., 2011). Briefly, MDCK cells were infected with 100 μL of 10-fold serial dilutions of sample and incubated at 37 °C for 72 h. Following incubation, viral titers were determined by hemaglutination assay using 0.5% turkey red blood cells and analyzed by the method of Reed and Meunch (1938).

### Literature search criteria

To investigate the known literature on the effects of statins on pneumonia outcome and etiology, a comprehensive search of the peer-review literature was conducted on PubMed based on a range of key terms including statin (or statin derivatives such as atorvastatin) and pneumonia, respiratory infection, influenza, and bacteria. Papers that met this criteria were then further refined by only choosing articles focused on human trials. Reviews were excluded from the search. These papers were then scrutinized for type of statin used, outcomes, presence of obesity or BMI as a factor in the analysis or outcomes as well as clinical diagnosis of etiology.

## Results

**Figure 1:**
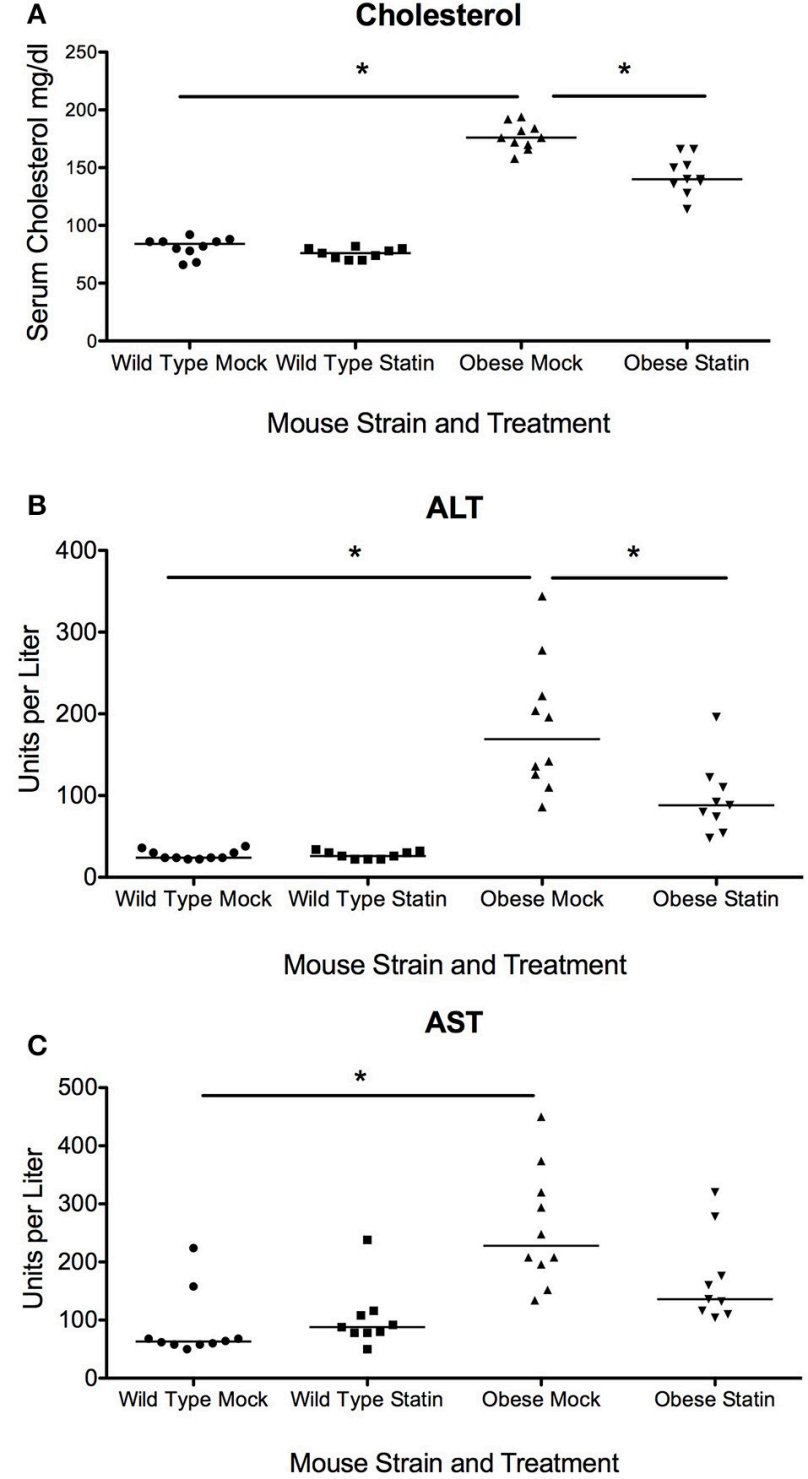
Effect of statins on total cholesterol and liver enzymes in wild-type and obese mice. **(A)** Total serum cholesterol in is elevated in obese mice compared to wild-type (*p* < 0.01, Mann-Whitney) and statin diet significantly reduces serum cholesterol in obese mice (*p* < 0.01, Mann-Whitney). **(B)** Serum ALT levels are elevated in obese animals and levels are significantly reduced in response to statin diet (*p* < 0.05, Mann-Whitney). **(C)** Serum AST levels are elevated in obese animals (*p* < 0.05, Mann-Whitney). Bonferroni correction was used to adjust for multiple comparisons. ^*^Indicates *p* < 0.05.

We hypothesized that that the effect of prophylactic statin therapy could have significantly altered benefit depending on the infectious agent, in this case bacterial and viral pneumonia, as well as the obesity status of the host. To test the impact of statins on acute respiratory infection in obesity, wild type and genetically obese mice were given either a statin or a mock diet for 4 weeks. Subsequent serum analysis confirmed a significant reduction in the levels of cholesterol in response to the statin diet, confirming pharmacological benefit (Figure 1A). In addition, benefit was observed in terms of liver function as measured by serum aspartate aminotransferase (AST) levels, though reduction in alanine aminotransferase (ALT) levels was not significant (Figures 1B,C). These data indicate that the statin intervention was effective at the doses administered with expected improvements in physiological parameters.

**Figure 2:**
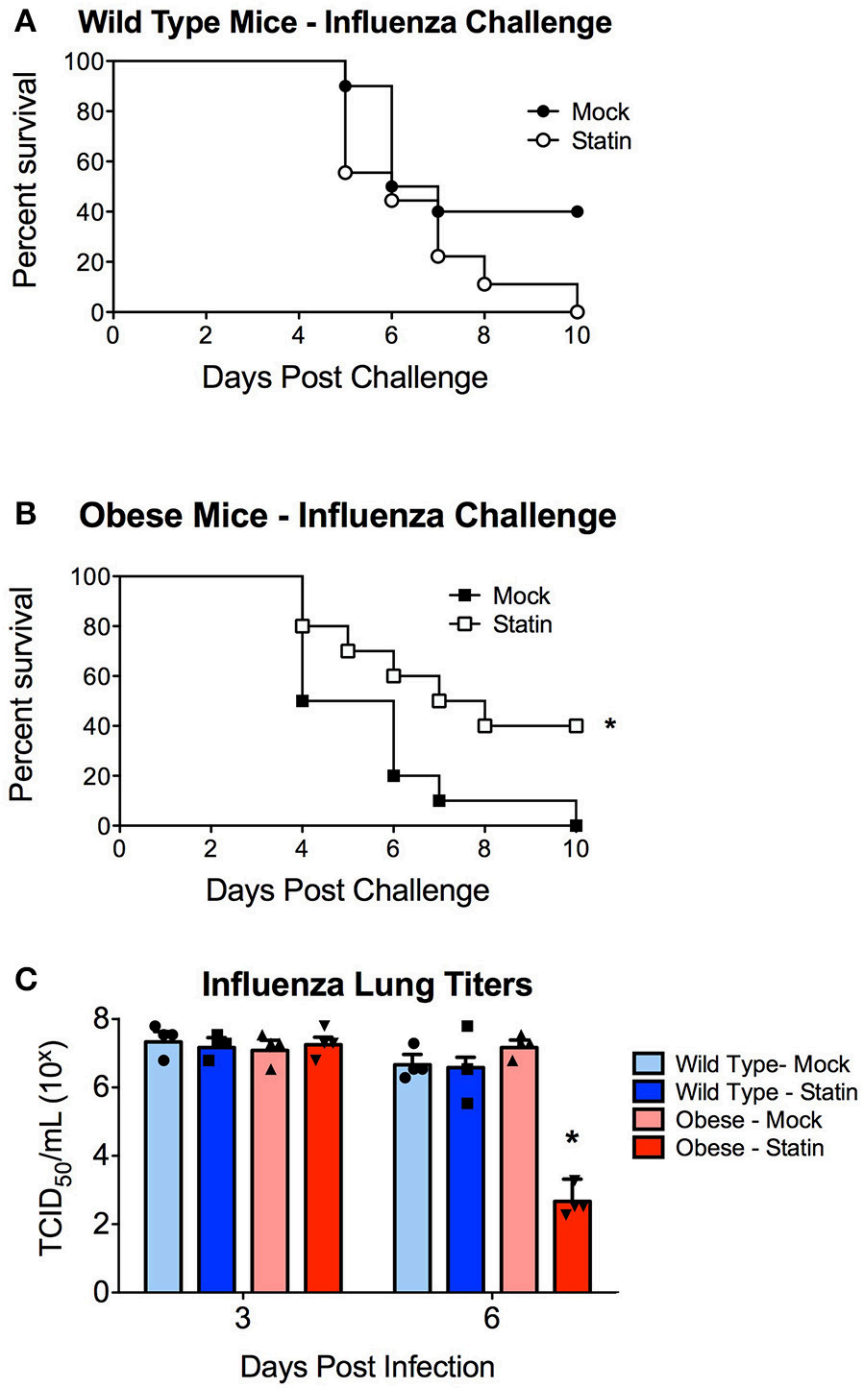
Impact of statin intervention on influenza pathogenesis in wild-type and obese mice. **(A)** Survival of wild-type mice receiving mock or statin supplemented diet following infection with influenza (*p* > 0.05 Mantel log-rank test). **(B)** Statin intervention significantly (*p* < 0.05 Mantel log-rank test) improved survival in obese mice following influenza infection. *n* = 10 mice per group. **(C)** Statin intervention significantly (*p* < 0.05, Mann-Whitney) decreased influenza viral burden in lungs 6 days post-challenge compared to mock treated animals. Note that statistical comparisons were made between each combination of two treatment groups to determine significance. ^*^Indicates *p* < 0.05.

We next sought to determine if statin intervention would alter the susceptibility of either wild-type or obese mice to respiratory infection. This was initially modeled using an influenza virus challenge. Obese mice were found to demonstrate enhanced susceptibility to influenza infection in concordance with previously published reports (Karlsson and Beck, 2010; Karlsson et al., 2017). Prophylactic statin intervention was found to confer no protective benefit against influenza induced mortality in wild-type animals (Figure 2A). In contrast, obese mice treated with statins were significantly less susceptible to influenza virus with a 40% increase in survival following infection (Figure 2B). Statin intervention was found to reduce influenza viral burden in the lungs 6 days post-infection though no alteration in viral titers at either earlier time points or in the wild-type animals in response to either mock or statin treatment was observed (Figure 2C). These data indicate that statin intervention may confer protective benefit in the context if influenza mediated pneumonia.

**Figure 3:**
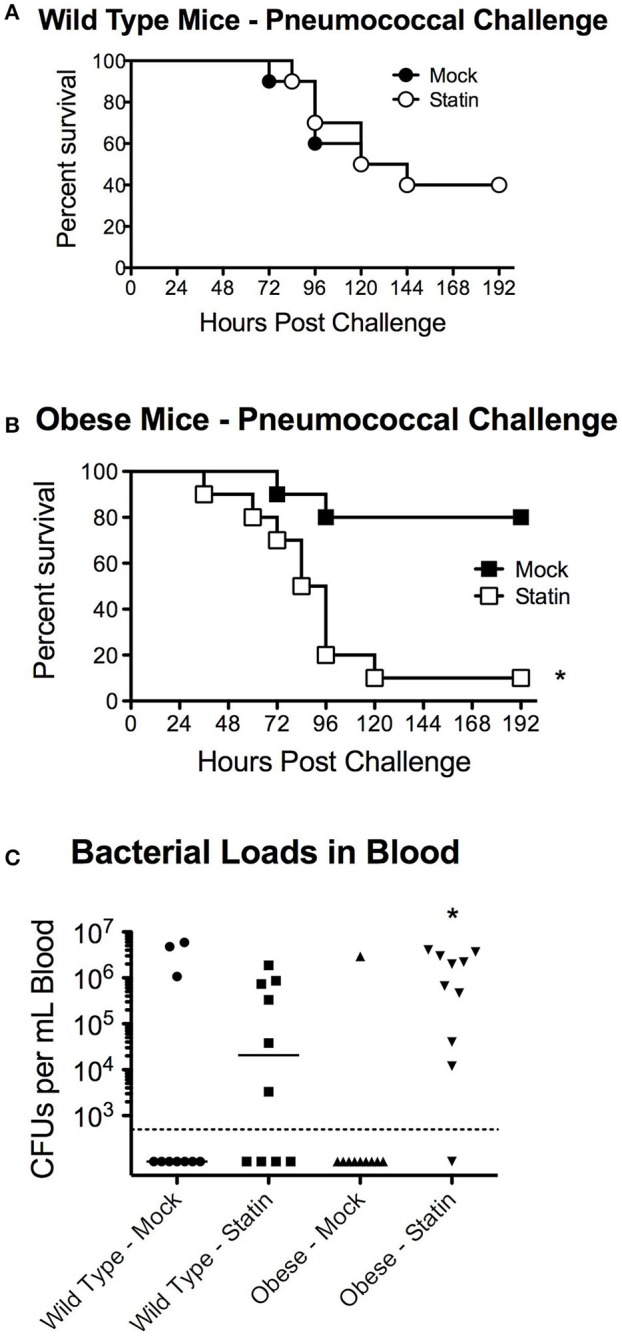
Impact of statin intervention on pneumococcal pathogenesis in wild-type and obese mice. **(A)** Survival of wild-type mice receiving mock or statin supplemented diet following infection with pneumococcus (*p* > 0.05 Mantel log-rank test). **(B)** Statin intervention significantly (*p* < 0.05 Mantel log-rank test) reduced survival in obese mice following pneumococcal infection. **(C)** Statin invention significantly increased bacterial burden in the bloodstream in obese animals (*p* < 0.05, Mann-Whitney). *n* = 10 mice per group. Note that statistical comparisons were made between each combination of two treatment groups to determine significance. ^*^Indicates *p* < 0.05.

We next sought to determine whether the metabolic-status dependent protection conferred by statin intervention could be extrapolated to other etiological agents of pneumonia, specifically pneumococcal pneumonia. In agreement with previous studies, no significant protection in terms of mortality was observed in wild-type statin treated animals compared to the mock group (Figure 3A). In contrast to the protective capacity observed in the influenza challenge models, obese mice receiving statins were significantly more susceptible to *S. pneumoniae* infection with a significant decrease in survival (Figure 3B). This altered survival was reflected by the relative pathogen burden, as statin treatment increased the level of bacteremia in the obese mice (Figure 3C). Collectively, these data demonstrate distinct responses in terms of mortality for statin-treated obese mice based on the etiology of the pneumonia that was not apparent in the mock treated animals. These data indicate that statin treatment has minimal effect in wild-type animals, but can potentially confer both a protective, in the case of influenza, and a detrimental, in the case of *S. pneumoniae*, effect on survival following infection.

**Figure 4:**
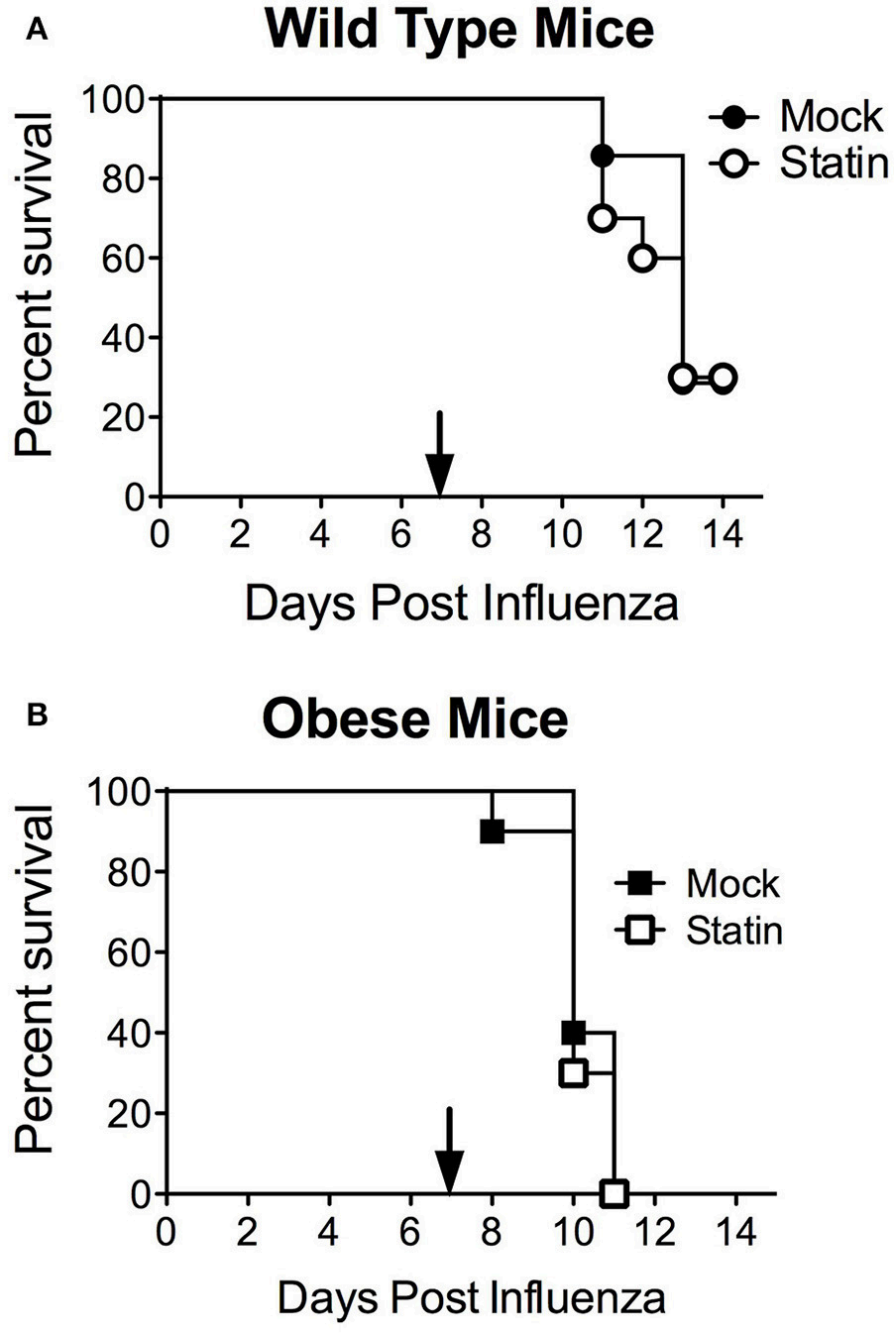
Impact of statin intervention in a model of influenza-pneumococcal co-infection. Statin therapy did not significantly improve or reduce mortality in either wild-type **(A)** or obese **(B)** mice (*p* > 0.05, Mantel log-rank test). *n* = 10 mice per group. Arrow indicates day of challenge with *S. pneumoniae*.

These data also call into the question the potential protective mechanism of statins in the context of influenza-mediated pneumococcal superinfection. It has been established both clinically and murine models that prior influenza infection greatly enhances host susceptibility to secondary bacterial infection, particularly with the pneumococcus through a variety of mechanisms (McCullers, 2014). We hypothesized that the opposing effects of statin therapy in obese animals in respect to bacterial and influenza challenge may be ameliorated in the context of co-infection. When mice were challenged in the co-infection model, neither protection nor enhanced susceptibility was observed in the influenza- *S. pneumoniae* co-infected mice, regardless of metabolic status (Figures 4A,B). These data indicate the opposing effects of statins in the context of obesity with viral or bacterial infection, by influenza and *S. pneumoniae* respectively, may cancel each other out in the context of co-infection.

**Figure 5:**
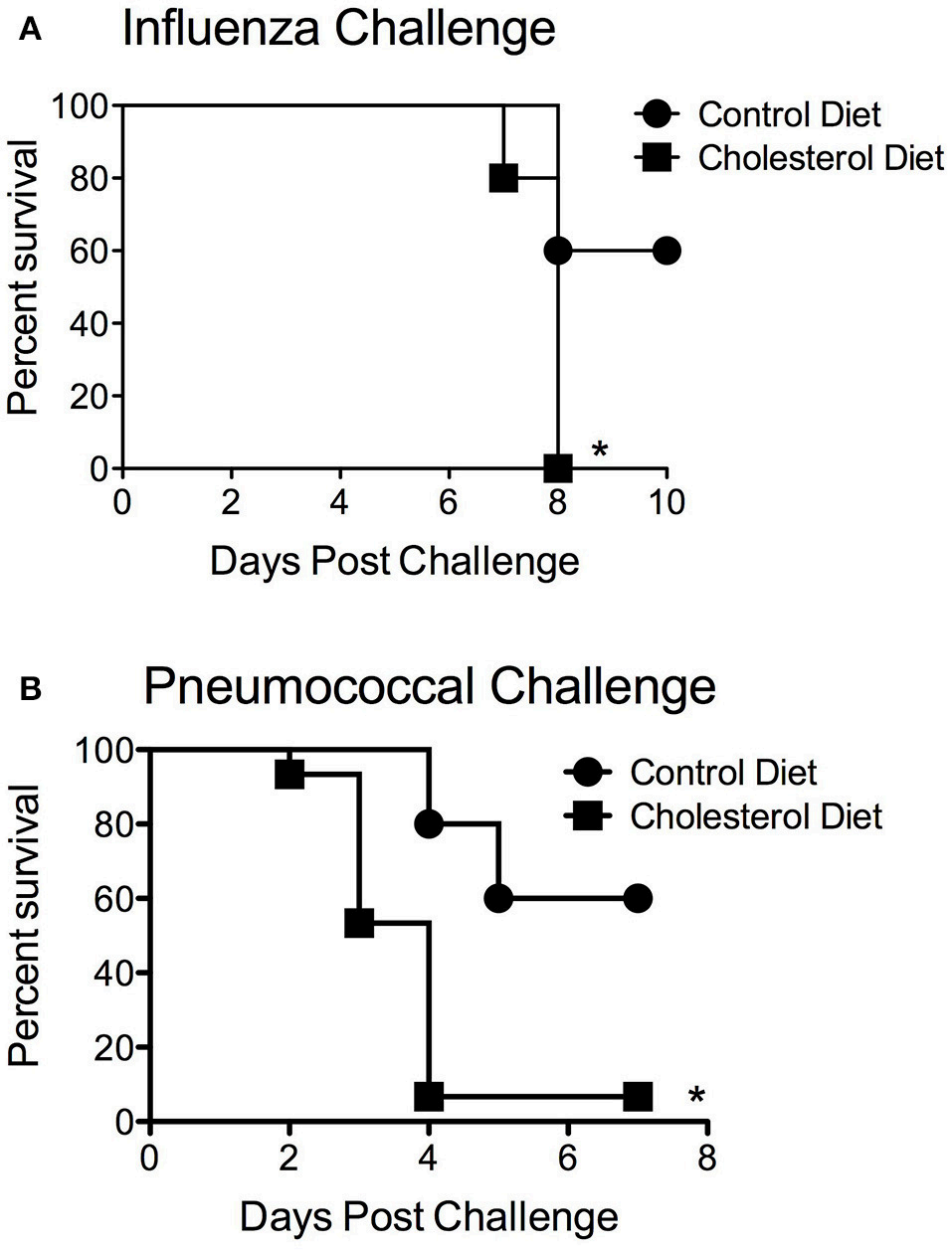
Impact of cholesteremia on infection susceptibility. Mice fed a high cholesterol diet had significantly reduced mortality (*p* < 0.05, Mantel log-rank test) compared to mice on the control diet in response to both influenza **(A)** and pneumococcal challenge **(B)**. *n* = 10 mice per group. ^*^Indicates *p* < 0.05.

There are several possible mechanisms that could contribute to the differences in statin-mediated alterations in obese but not wild-type animals. First of all, the lipid-altering effects of statins could be contributing to altered disease susceptibility. This is particularly relevant to the pneumococcus, as one of its major virulence factors is a cholesterol dependent cytolysin, pneumolysin, which could potentially be inhibited by high levels of serum cholesterol. To test this hypothesis, mice fed a high cholesterol diet, but which have a normal BMI, were infected with pneumococcus. The pneumococcal infection susceptibility was directly opposite from the obese phenotype with mice fed the high cholesterol diet being extremely susceptible to bacterial infection (Figure 5A). In contrast to the obese animals, the high-cholesterol diet mice demonstrated heightened susceptibility to both *S. pneumoniae* and influenza infection (Figure 5B). These results are in support of previous findings that serum cholesterol levels may have an inverse relationship with influenza infection (Iribarren et al., 1997). These results are in contrast to the susceptibility of the obese animals to *S. pneumoniae* infection. This suggests factors independent of cholesterol levels are likely mediating the differences in susceptibility to infection in the context of statin prophylaxis in the obese murine models.

## Discussion

The pleiotropic effects of statins with potential protective effects extending to pneumonia and sepsis have long been recognized. These effects have primarily been ascribed to their proposed immune modulatory functions, though the clinical data has been conflicted into the potential protective benefit despite large patient cohorts (Table 1 and reference therein). The inherent variability of patient populations on statin intervention, in particular metabolic status, could be a major confounding factor that many studies do not incorporate despite an accumulating amount of evidence of the impact of obesity on immune responses and infection susceptibility, particularly in the context of influenza infection (Green and Beck, 2017). This heightened infection risk in obese individuals is apparent despite current vaccination efforts (Neidich et al., 2017). Further confounding the situation is that diagnosing the etiological agents of pneumonia can be particularly challenging with considerable institutional variance (Murdoch et al., 2009). These confounders can be difficult to access from clinical data, as discerning between viral and bacterial pneumonia from chest x-rays can be challenging (Graffelman et al., 2008). As such, the responsible infectious agents in such large clinical cohorts as many of the statin-related studies have compromised have been limited to sepsis and pneumonia without further discrimination based on the responsible pathogen.

**Table 1:** Examples of the role of statin therapy on pneumonia.

**Year**	**Statin used**	**# Participants**	**Protection**	**Bacterial vs. Viral**	**Obesity status**	**References**
2008	Non-specific	5,772	Y	N	N	Mortensen et al., 2008
2009	Non-specific	3,681	Y	N	N	Myles et al., 2009
2008	Simvastatin, atorvastatin, pravastatin	1,007	Y	N	N	Chalmers et al., 2008
2011	Non-specific	129,288	Y	N	N	Douglas et al., 2011
2013	Non-specific	347	Y	Viral only	N	Doshi et al., 2013
2012	Non-specific	22,996	Y	N	N	Mortensen et al., 2012
2009	Simvastatin, lovastatin, atorvastatin	1,125	N	N	N	Dublin et al., 2009
2007	Non-specific	76,232	Y	Y	N	Frost et al., 2007
2012	Non-specific	121,254	Y	Y	Y	Rothberg et al., 2012
2007	Non-specific	1,253	Y	N	N	Schlienger et al., 2007
2011	Atorvastatin, simvastatin	17,755	Y	N	N	Vinogradova et al., 2011
2008	Simvastatin, atorvastatin, pravastatin	29,900	Y	N	N	Thomsen et al., 2008
2011	Simvastatin, atorvastatin, pravastatin, lovastatin, fluvastatin	1,895	N	N	N	Yende et al., 2011
2012	Simvastatin, atorvastatin, pravastatin	7,223	Y	N	Y	Nielsen et al., 2012
2012	Rosuvastatin	17,802	Y	N	Y	Novack et al., 2012
2011	Pravastatin	152	Y	N	N	Novack et al., 2012
2006	Non-specific	4,719	Y	N	N	van de Garde et al., 2006
2005	Non-specific	787	Y	N	N	Mortensen et al., 2005

The observation that statins were beneficial for a viral infection but detrimental for a bacterial infection in the context of obesity was an unexpected finding. Obesity is known to impact of number of immunologic processes though the mechanisms underlying these differences are mechanistically complex (Milner and Beck, 2012). One potential explanation is that the statins alter the inflammatory state as to favor the progression of bacterial but not viral pneumonia in obese individuals. During the pneumococcal pneumonia the inflammatory response consists of a rapid recruitment of neutrophils to mediate clearance of the bacteria and is an extremely rapid, acute response. Obesity can dramatically impact neutrophil migration through leptin signaling as well as impact neutrophil oxidative capabilities (Gainsford et al., 1996; Caldefie-Chezet et al., 2001, 2003). Leptin signaling in obesity has also been associated with the production of numerous pro-inflammatory cytokines, whose signaling play important roles in the initial phases of controlling pneumococcal pneumonia (Matarese et al., 2005). As such, if statins were decreasing production of such inflammatory cytokines, one might expect to observe heightened susceptibility to pneumococcal infection due to reduced initial bacterial clearance in obese animals.

In contrast to pneumococcal infection, mortality for the influenza infection typically occurred days later indicating this response may be more dependent more upon the adaptive immune response. It has been demonstrated that leptin can enhance for the proliferation and the activation of T-cells, though such stimulation was dependent upon a stimulatory co-factor (Martín-Romero et al., 2000). Leptin was also found to enhance the production of pro-inflammatory cytokines by T-cells as well as limit apoptosis in response to stress (Fujita et al., 2002; Lord et al., 2002). Simvastatin has been shown to modulate T-cell responses in models of airway inflammation such as asthma, but requires accessory cells for the observed effects (Liu et al., 2014; Knobloch et al., 2016). This modulation of adaptive immunity by statins in the context of obesity may play an important role in the observed protective effects observed in these animals.

Overall, these results indicate the need for controlling for both infection etiology as well as risk factors such as obesity during epidemiological studies to determine the groups who will benefit or possibly have increased risk of respiratory infection from the use of statins. Further work on the effects of statins on the pathogenesis and immunological responses against primary and co-infection with respiratory pathogens is warranted.

## Author contributions

EK, SS-C, and JR designed the experiments, interpreted the data, and wrote the manuscript. EK and JR performed the experiments.

### Conflict of interest statement

The authors declare that the research was conducted in the absence of any commercial or financial relationships that could be construed as a potential conflict of interest.
